# Chemical Composition, Antioxidant Activity, Cytoprotective and In Silico Study of Ethanolic Extracts of *Bougainvillea* × *buttiana* (Var. Orange and Rose)

**DOI:** 10.3390/molecules27196555

**Published:** 2022-10-03

**Authors:** Vera L. Petricevich, Mayra Cedillo-Cortezano, Rodolfo Abarca-Vargas

**Affiliations:** Facultad de Medicina, Universidad Autónoma del Estado de Morelos (UAEM), Cuernavaca 62350, Mexico

**Keywords:** chemical compounds, antioxidant activity, cytoprotective effect, in silico study, *Bougainvillea* × *buttiana*

## Abstract

*Bougainvillea* × *buttiana* is a plant widely used in traditional Mexican medicine and other parts of the world for the treatment of various health disorders. In this study, the antioxidant and cytoprotective activities of three ethanolic extracts of *B*. × *buttiana* (BxbO (Orange), BxbR1 (Rose1) and BxbR2 (Rose2)) were investigated. Antioxidant activities were determined by the oxygen radical absorbance capacity (ORAC), DPPH free radicals scavenging activity, and radical scavenging effects on nitric oxide (NO). The in vitro cytoprotective effect of the extracts against oxidative stress induced by hydrogen peroxide-(H_2_O_2_) in a model of L929 cells was also determined as well as NO uptake with or without H_2_O_2_ through the MTT assay. The results revealed that there was a difference between the compounds present in each of the extracts, with the 2-Hydroxycinnamic acid compound being observed in all the extracts. The 2-Hydroxycinnamic acid compound was tested in silico to predict its biological (PASSonline) and toxicological (Osiris Property Explorer) activity. All extracts with 1 to 4 mg/mL inhibited the activity of the NO radical. In cells exposed to 1 mg/mL of extracts followed by H_2_O_2_ exposure, cell protection ranged from 66.96 to 83.46%. The treatment of the cells with extracts prevented the morphological changes caused by H_2_O_2_. The 2-Hydroxycinnamic acid compound showed a probability of in silico antioxidant and cytoprotective activity greater than 0.5 and 0.6, respectively. Therefore, the results demonstrated that Bxb extracts exert antioxidant and protective activities against H_2_O_2_-induced oxidative stress in L929 cells.

## 1. Introduction

Oxidative stress is an imbalance between oxidative and antioxidant molecules and results in the induction of cellular damage by oxidants. This imbalance occurs when the mechanisms of protection against these species are impaired or when a high production of reactive oxygen species (ROS) and/or reactive nitrogen species (RNS) occurs [1]. With a slight oxidative stress, it can generate an increase in the enzymatic antioxidant protection; however, when a high production of reactive species occurs, it can cause damage and cell death [2]. Biomolecules oxidations are natural processes in aerobiotic beings from the cellular metabolism, in which reactive species are produced naturally or by a some biological dysfunction [3]. This is an integral part of the human metabolism to produce ROS and/or RNS, in which reactive species have important biological functions, such as phagocytosis, but when their production is exacerbated, the organism has an efficient antioxidant system in which it can control or restore the equilibrium [4]. An excess of reactive species is harmful to the organism, and it can cause, for example, the peroxidation of membrane lipids and the aggression of the macromolecules of the tissues. The literature indicates the possibility that chronic degenerative diseases, such as diabetes mellitus, cancer, early cell aging, atherosclerosis, Alzheimer’s and Parkinson’s, disease, are related to DNA damage caused by the species reactive as well as oxidative stress [5]. According to their mode of action, the antioxidants can be categorized as primary or secondary. The primary antioxidants are those that act in the interruption of the reaction chain by donating electrons or hydrogen to the free radicals to convert them into thermodynamically stable products and/or react with the free radicals, which will form a lipid antioxidant, which can react with another free radical. On the other hand, secondary antioxidants act by delaying the onset of autoxidation, in different ways, among them metal complexation, oxygen sequestration, the decomposition of hydroperoxide forming non-radical species, the absorption of ultraviolet radiation and the deactivation of singlet oxygen [6,7].

Free radicals are chemical structures that have one or more free electrons occupying an atomic or molecular orbital [7,8]. Due to their configuration, free radicals have highly unstable, short half-lives and are chemically unstable, combining nonspecifically with numerous molecules of the cellular structure [8]. The formation of free radicals takes place through the absorption of radiation by redox reactions or by processes of enzymatic catalysis [8,9]. Free radicals are present inside the cellular metabolism of living beings and are generated naturally during oxidation-reduction approaches, which include the production of energy, cell growth regulation, intracellular signaling, phagocytosis and biomolecules synthesis [10]. The process of electron transfer can lead oxygen to form reactive oxygen species and/or reactive nitrogen species [11]. The main reactive oxygen species are superoxide anion radicals (O_2_^−^), hydroxyl radicals (OH^−^), hydrogen peroxide (H_2_O_2_), singlet oxygen (½O_2_^−^), hypochlorous acid (HOCl), nitric oxide (NO) and peroxynitrite (ONOO^−^) [10,11]. The superoxide dismutase (SOD) family is responsible for converting superoxide into molecular oxygen and hydrogen peroxide. H_2_O_2_ can be converted to OH^−^, which is a highly reactive species, and this reactive species can also react with nitric oxide (NO), resulting in the formation of peroxynitrite (ONOO^−^). Nitric oxide generation was associated with caspase activation and cell death [1,11]. NO is a gaseous, small, reactive molecule that readily diffuses across the cells and interacts with different cellular compounds, including other radicals [12]. Nitric oxide and ROS are involved and interact with each other in a wide range of cellular processes [13,14]. Due to the high complexity, this process is still unclear. The multiple physiological functions of NO include the inhibition of platelet aggregation, the regulation of vasomotor tone, endothelial cell adhesion, and the proliferation of vascular smooth muscles [15,16]. Biologically produced NO is also important for nonspecific cellular immunity, although NO is not capable of killing intracellular pathogens or tumor cells. Although often described as a highly toxic and reactive molecule, it has been shown not to be so and to present a dual cytotoxic and cytoprotective [17,18].

Previous in vitro and in vivo studies have reported the antioxidant capacity of several species of medicinal plants of the Nyctaginaceae family [19], acting at the cellular level through cell growth stimulation and membrane potential stabilizing or at the molecular level through ROS scavenging [17,20]. Plants are sources of bioactive compounds with antioxidant action such as phenolics, flavonoids and tannins that act in the capture and neutralization of oxidant species such as superoxide anions, hydroxyl radicals and hydrogen peroxide [18]. Endogenous antioxidants are essential against free radicals and oxidative damage. In case of endogenous antioxidants from plants, some have been widely used. In plants, the phenolic acid possess a different biological activity [17,18,21]. Traditionally, dried flowers of *Bougainvillea* are used for topical treatments of contusions and pain [21]. Despite their traditional use, no study has reported on the cytoprotective activity of *Bougainvillea* extracts in a cell line of mouse fibroblasts. In previous studies, we demonstrated that the antioxidant activity of extracts of *B*. × *buttiana* [22]. The aim of this study was to compare the chemical composition of BxbO, BxbR1 and BxbR2 (Varieties Orange, Rose1 and Rose2), respectively, their antioxidant and cytoprotective activities, by predicting both activities in silico studies. The in vitro antiradical activity was investigated using a NO scavenging assay.

## 2. Materials and Methods

### 2.1. Chemicals

Ethanol was acquired from Golden Bell (Zapopan, Jalisco, Mexico). Actinomycin D, O-phenylenediamine (OPD), 4-nitro-(3-octanoyloxy)-benzoic, phosphoric acid, 2,2′-azino-bis(3-ethylbenzothiazoline-6-sulfonic acid) (ABTS), sodium nitroprusside, and 3-[4,5-dimethyl-thiazol-2-yl]-2,5-diphenyltetrazolium bromide (MTT) were purchased from Sigma Aldrich Chemical Co. (Toluca, Mexico). Dulbecco’s Minimum Essential Medium (DMEM) medium fetal calf serum was acquired from Gibco Life Technologies Corporation (Grand Island, NY, USA).

### 2.2. Plant Material Collection and Extraction

#### 2.2.1. Plant Material Collection

The specimens of the *B*. × *buttiana* plant were collected in Cuernavaca and Temixco (Morelos, Mexico), and identification from the specimen was made by Herbarium HUMO CIByC-UAEM and cataloged with the voucher number 23683 (Cuernavaca), 33870 (Cuernavaca), and 33872 (Temixco), for varieties Orange (BxbO), Rose1 (BxbR1) and Rose2 (BxbR2).

#### 2.2.2. Extraction Procedures

The extraction method was reported in detail in the patent MX/a/ 2011/813522 [22]. For different extraction, 10 g of powered BxbO, BxbR1 and BxbR2 were individually mixed with 100 mL of ethanol + water (1:1) at 26 °C for 24 h. Liquids extracts obtained were separated from the solid residue by 60 °C using a rotary evaporator pressure (Heidolph). All samples were performed in triplicate. Extracts from each extraction process were collected in separate extraction vials and maintained at room temperature until the phytochemical assays.

#### 2.2.3. Analysis of Total Phenolic Contents

The total phenolic contents present in the extracts of BxbO (Orange), BxbR1 (Rose1) and BxbR2 (Rose2) were determined by the Folin–Ciocalteu assay using a gallic acid standard curve [23]. The absorbance determination was at 760 nm using a UV-Vis spectrophotometer, and the results were expressed as mg gallic acid equivalents (GAE) per 100 g FW. The equation is shown below:

(1) $$C=\frac{c\;\times \;V}{m}$$

where “C” indicates the total phenolic component in (mg g^−1^) plant extract in GAE, “c” indicates the gallic acid concentration (mg mL^−1^), “V” indicates the volume of extracts in microliters (µL), and “m” indicates the weight of crude plant in grams.

The correlation coefficients (R^2^) value was determined using the mean of three absorbance determinations for each concentration. The equation is shown below:

(2) Y=mx+c

where “Y” signifies extract absorbance, “m” signifies the slope of the calibration curve, “x” signifies extract concentration, and “c” is the intercept. Concentrations of extracts were calculated using this regression equation. The phenolic content was estimated using the value for each extract concentration.

#### 2.2.4. GC-MS Analysis for Characterization

For the identification, the extracts of *B*. × *buttiana* (Var. Orange and Rose 1 and Rose 2) they were called BxbO and BxbR1 and BxbR2, respectively. The analyses of each extract were performed with a chromatography GC-MS method. The analyses were performed according to the method described by Abarca-Vargas et al. 2016 [24].

### 2.3. Measurement of Antioxidant Activity

#### 2.3.1. ORAC Oxygen Radical Absorbance Capacity

The antioxidant activity was determined using Oxygen Radical Absorbance Capacity (ORAC) assay, as described by Dávalos et al., 2004 [25]. In brief, twenty-five microliters of Trolox standards (ranging from 3.125 to 100 µM), blank and sample of extracts were prepared in phosphate buffer 10 mM and pH 7.4. The reaction mixture containing extracts or Trolox and fluorescein at a final concentration of 70 nM was incubated for 10 min at 37 °C. Then, 12 mM of 2,2′azobis(2-amidno-propane) dihydrochloride was added to the mixture. The fluorescence was read every 56 s for 98 min by using FLUOstar Optima (BMG Labtech) fluorometer. The Area Under the Curve (AUC) was determined for each sample and compared with AUC corresponding Trolox. The data were expressed as an ORAC value (µmol Trolox equivalent (TE)/g extract).

#### 2.3.2. DPPH Radicals Scavenging Activity

The antioxidant activity was determined using DPPH radical scavenging assay as adapted by Akkari et al., 2016 [26]. In brief, the volume of 1.4 mL solution of DPPH in methanol (0.1 mM) was added to 0.1 mL of each extract with different concentrations (10, 20, 40, and 80 × dilutions of each extract). Then, the tubes were incubated in a dark room for 30 min, and the absorbance was determined at 517 nm.

#### 2.3.3. Antioxidant Activity NO (Nitric Oxide) Assay

The nitric oxide capture assay was performed according to the method described by Maia & Moura, 2015 [27]. The assay was performed in PBS (10 mM phosphate buffer, pH 7.3, 136 mM NaCl, and 2.7 mM KCl) and 5 mM sodium nitroprusside. Amounts from each extract (0, 0.001, 0.01, 0.1, 1 and 2 mg/mL) were added in a nitroprusside solution and incubated in a shaker at 25 °C with constant shaking at 70 rpm for 180 min. Gallic acid was used as standard in the concentration range of 0–160 µg/mL to construct a calibration curve. The control used for these assays presented a 100% nitrite formation. After incubation, an aliquot was removed and transferred to a 96-well plate and incubated with Griess’s reagent (1% sulfanilamide solution in 3% phosphoric acid and 1% N-1 (1-naphthylethylenediamine) solution). The microplates containing the assays were maintained for 5 min at room temperature, and the absorbance was evaluated on a microplate reader at 540 nm. A calibration curve of sodium nitrite at concentrations of 0.0265 to 1 mM was performed to determine the nitrite concentrations present in the assays. The results were expressed as the mean standard deviation in percent inhibition relative to the control assay. All tests were run in sets of three plus the standard. The results were expressed by the percentage of antioxidant activity and were calculated using the following formula:

(3) $$\mathrm{AA}(\%)=\frac{A_{control}-A_{sample}}{A_{control}}\times 100$$

where *A_sample_* represents absorbance of the plant extract sample and *A_control_* represents absorbance of the DPPH solution as a control.

### 2.4. Cell Proliferation and Viability Assay

Mouse fibroblast cells (ATCC clone L929) were grown in Dulbecco’s Minimum Essential Medium (DMEM) containing 10% Fetal Bovine Serum (FBS), 100 U/mL penicillin, 100 mg/L streptomycin and 500 mg/L neomycin. Confluent cells were tripsinized, centrifuged and sub-cultured in the same medium, in a humidified 5% CO_2_ atmosphere at 37 °C. For the experiments, a cell suspension was seeded at a density of 5 × 10^3^ cells/well in DMEM culture medium supplemented with 10% FBS and dispensed in a 96 well microplate and incubated at 37 °C in a humidified 5% CO_2_ atmosphere for 18 h. Afterwards, the culture medium was discarded and replaced with fresh medium containing 10% FBS for the control cultures, and amounts between 0 and 400 µg/mL of dry weight of each extract were distributed to each well. All cultures, including the controls and those treated with the extracts, were incubated for 24 h and 48 h at 37 °C under 5% CO_2_ atmosphere. Then, the culture medium was discarded and finally restored with 100 µL of fresh medium combined with 10 µg of MTT solution 3-(4,5-dimethylthiazol-2-yl) 2, 5-diphenyltetrazolium bromide and keep within dark conditions at 37 °C in a 5% CO_2_ atmosphere for 3 h. After that time, 85 µL of the culture medium was removed, and then, a 50% solution of DMSO was added and incubated under the same conditions for 10 min; after formazan crystals’ homogenization, the absorbance at 540 nm was assessed using a microplate reader. The percentage of cell proliferation/viability was calculated and compared to the control (100% viability).

### 2.5. Hydrogen Peroxide-Induced Oxidative Stress in L929 Cells and Evaluation of Survival

To evaluate the cytoprotective effect of extracts, hydrogen peroxide was used to induce oxidative stress assay in accordance with the methods previously described by Balekar et al. 2012, [28]. Briefly, the L929 cells were cultivated at a density of 5 × 10^3^ cells/well in DMEM supplemented with 10% FBS and incubated at 37 °C with 5% CO_2_ for 18 h. To establish the H_2_O_2_ concentration that provided cell damage, amounts of 0 to 1.0 mM were used for the hydrogen peroxide curve to evaluate the dose of H_2_O_2_, which caused a decrease of 50–80% in the cell viability after 24 h of exposure using the MTT method. The concentration chosen was 1.0 mM H_2_O_2_. For the assays, the L929 cells were cultured at a density of 5 × 10^3^ cells/well containing DMEM medium supplemented with 10% FBS and incubated at 37 °C with 5% CO_2_. After 18 h of incubation, the medium was replaced with fresh medium containing 0 to 400 µg/mL dry weight of *B*. × *buttiana* extracts (Var. Orange and Rose), which were used to treat the cells at the different time periods.

### 2.6. In Silico Analysis of the Compounds

All chemical compounds identified in this study were represented by using their chemical structure, which was obtained using the ChemAxon program [29]. Predictions of all compounds were determined using the chemical structure. PASSonline software (Way2Drug.com, 2011–2022, version 2.0, accessed 1 August 2022) [30] was used to predict antioxidant and cytoprotective activity. This program calculates the structural and physicochemical properties necessary to make a comparison with its database. These results are expressed as a probability (P) ranging from 0 to 1, where 0 indicates that it is unlikely and 1 that it is very likely.

The in silico toxicological properties were also determined using the OSIRIS Property Explorer program [31]. This software allows calculating the properties without risk of unwanted effects and is shown in green, while those with medium risk are indicated in yellow, and in the case of high risk, they are represented in red.

### 2.7. Statistical Analyses

The data were obtained for each sample and summarized, which was followed by statistical analysis using one-way ANOVA and Tukey’s test. Values of *p* lower than 0.05 were considered to be statistically significant. The values were expressed as the mean ± SD. Pair-wise comparison of the control and each sample was carried out using a t-test. Significant statistical differences were considered at *p* < 0.01 compared to the untreated control.

## 3. Results

### 3.1. Phytocompounds

**Figure 1:**
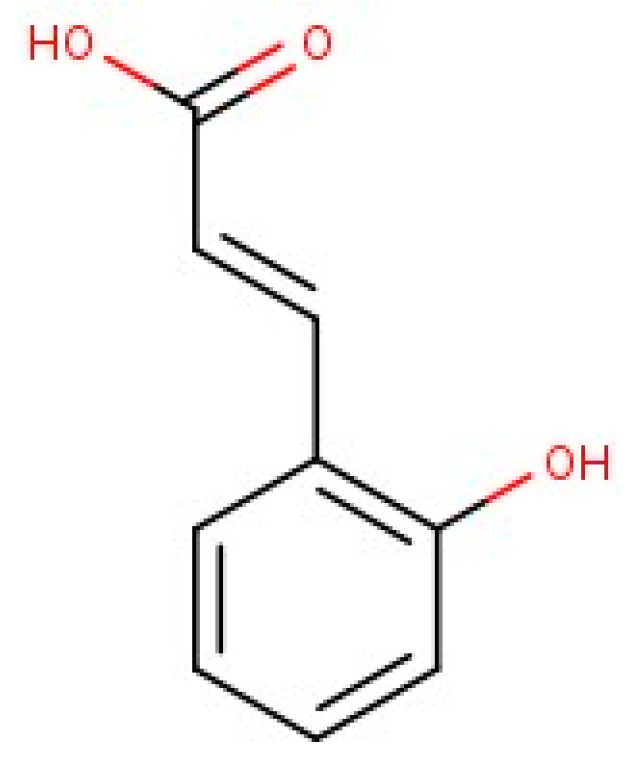
Chemical structure of 2-Hydroxycinnamic acid.

Each plant was extracted in 50% ethanol (*v/v*) under the same conditions. GC-MS analyses of these extracts showed several peaks for BxbO (nine compounds), BxbR1 (seven compounds) and BxbR2 (eight compounds). Table 1 summarizes the compounds present and analyzed by chromatography GC-MS [32,33,34]. They were quantified by integration of the peaks areas, and the results are exhibited in Table 1. Between the compounds obtained, the 2-Hydroxycinnamic acid and 3-O-methyl-D-glucose were present in all extract studies. The chemical structure of the compound 2-Hydroxycinnamic acid (Figure 1) was made with the ChemAxon version 22.13.0, (Budapest, Hungary) program [29].

### 3.2. Total Phenolic Contents in Bougainvillea Extracts

The Total Phenolic Contents (TPC) in *B. × buttiana* ethanolic extracts are presented in Table 2. The highest amounts of TPC were obtained in BxbR2 extract. The TPC ranged from 27.43, 29.55 and 32.48 mgGA/g dry extract for BxbO, BxbR1 and BxbR2, respectively.

### 3.3. Comparative Antioxidant Activity

The antioxidant activity of different extracts was evaluated by measuring their ability to scavenge DPPH and by ORAC assay. As shown in Table 3, the DPPH radical scavenging activities of three extracts followed the order of BxBR2 > BxbR1 > BxbO. Regarding the ORAC assay, BxbR1 and BxbR2 improve the ORAC values to 8100.45 and 8105.38 µmol Trolox/mg gallic acid, respectively. The lowest ORAC value was obtained in the BxbO extract. Results of antioxidant capacities were also correlated to phenolic compound concentration determined by the Folin–Ciocalteu method. Results were obtained with DPPH and ORAC assay.

### 3.4. Effect of Extracts on Nitric Oxide Capture

**Figure 2:**
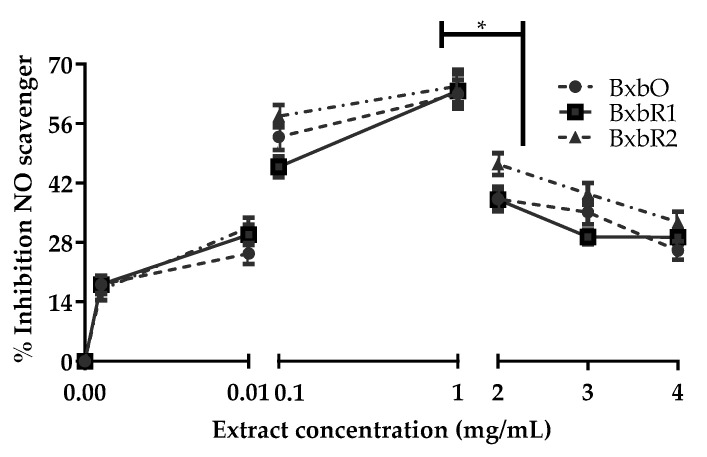
Inhibitory effect of *Bougainvillea* extracts on nitrite production (NO_2_^−^). The extracts in mg of dry mass per mL of assay. The percent inhibition of nitrite formation in the presence of extracts in relation to control with 180 min of reaction. All values are expressed as the mean ± SD (*n* = 3). The * indicate significant differences (*p* < 0.01).

The antioxidant activity of ethanolic extracts of *B*. × *buttiana* was determined using a NO scavenging assay. Figure 2 shows the nitrite concentration values and the percentage of inhibition of the formation of this anion by increasing the concentrations of the *Bougainvillea* extracts. The concentration of nitrite in the absence of the extract was approximately 26.2 μM. The formation of nitrite occurs spontaneously in the reaction medium from NO and O_2_, being an indirect method of determination of NO generated by the decomposition of sodium nitroprusside [27]. The extracts of BxbO, BxbR1 and BxbR2 at the concentrations used (0.001 and 0.01 mg/mL) were shown to be able to decrease the formation of nitrite, differentiating significantly in relation to the control but without a significant difference between them. The inhibition of NO increased with the use of extract concentrations of 0.1 mg/mL, reaching the maximum with 1 mg/mL concentration of the extracts. At concentrations of 1 mg/mL, the inhibition NO percentages was 62.97, 63.75 and 64.85% for BxbO, BxbR1 and BxbR2, respectively. In contrast, when concentrations of 2 to 4 mg/mL were used, the NO inhibition was significantly reduced (*p* < 0.001). These results suggest that extracts of BxbO, BxbR1 and BxbR2 act as antioxidants against NO (Figure 2).

**Figure 3:**
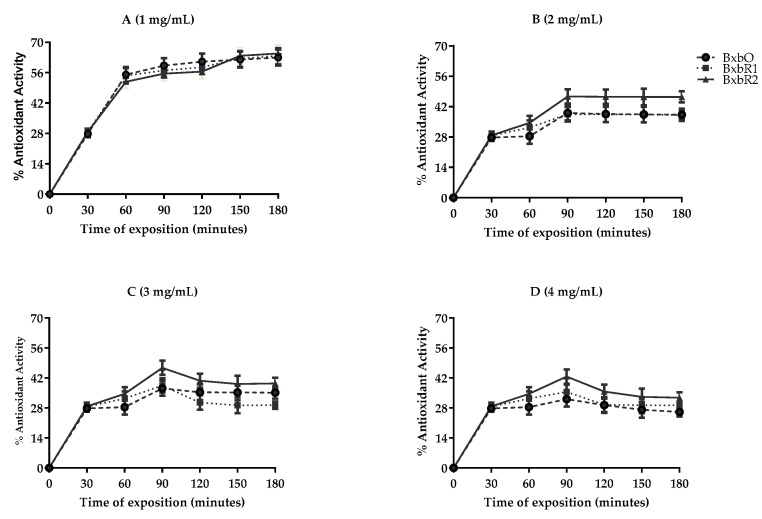
Percent of antioxidant activity. The inhibitory effect of *Bougainvillea* extracts on nitrite production (NO_2_^−^). The extracts in (**A** 1 mg), (**B** 2 mg), (**C** 3 mg), and (**D** 4 mg) of dry mass per mL of assay. The percent antioxidant activity inhibition of nitrite formation in the presence of extracts in relation to the control incubated for different times of reaction. All values were expressed as the mean ± SD (*n* = 3). This indicates values were significant differences (*p* < 0.01).

The kinetics of antioxidant activity is described in Figure 3. For the positive control for nitrite inhibition, gallic acid was used, reaching the highest percentage of antioxidant activity (48.84%) with 80 µg/mL incubated for 180 min (data not shown). The formation of nitrite decreased with increased exposure time to the extracts. The maximum antioxidant activity percentages reached by the extracts at 1 mg/mL were 62.70, 63.75 and 64.85% for BxbO, BxbR1 and BxbR2, respectively (Figure 3). With increasing concentrations at 2 to 4 mg/mL of extracts, the percentages of antioxidant activity were significantly reduced.

### 3.5. Effect of Extracts on Cell Viability and Proliferation

**Figure 4:**
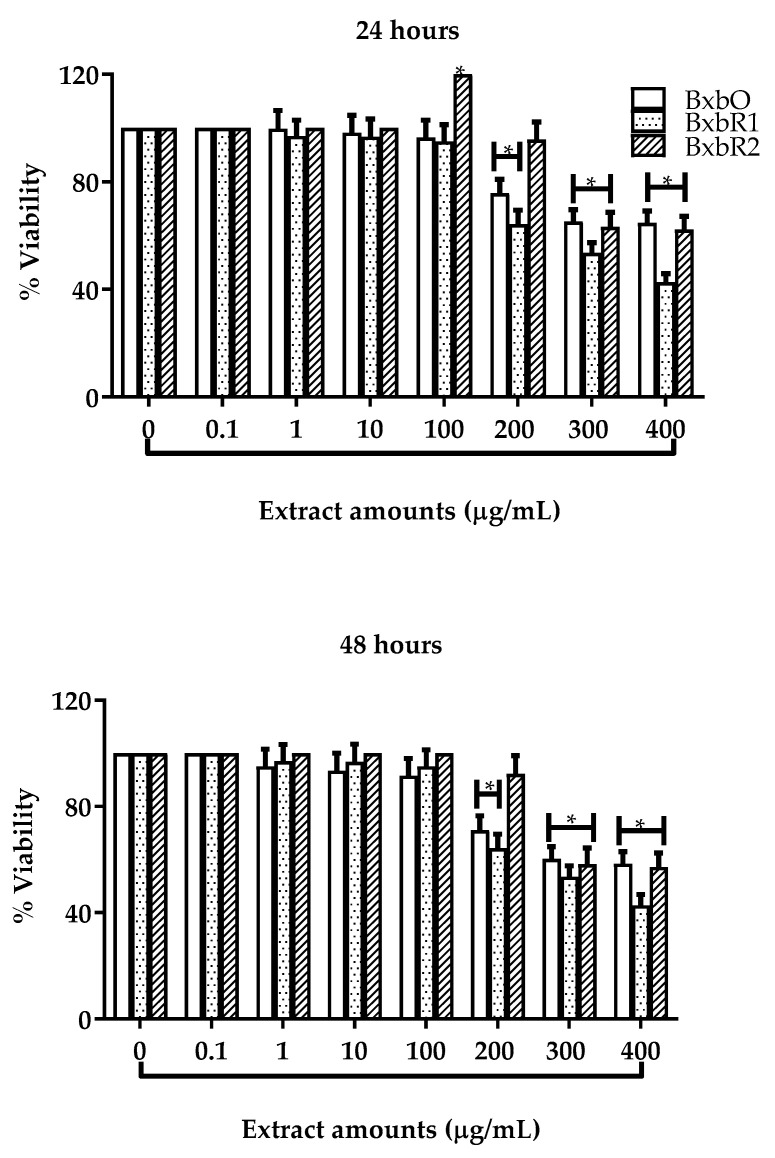
Effects of the extracts from *B*. × *buttiana* on the cell viability. Percentage of survival of L929 cells treated with extracts after 24 and 48 h. The data are expressed as the mean ± SD (*n* = 20). * *p* < 0.001.

The effect of the extracts from *B*. × *buttiana* using Var. Orange and Rose on the viability was evaluated in L929 cells in different concentrations after 24 and 48 h using the MTT method (Figure 4). Cultures of cells were treated with 0.1, 1 and 10 µg/mL extracts, and the extracts did not prove to be toxic to the cells. For cultures treated for 24 h at 100 µg/mL, the percentages of cell viability were 96.4, 95 and 120% for the extracts BxbO, BxbR1 and BxbR2, respectively. Whereas, in cultures treated for 48 h at 100 µg/mL, the percentages of cell viability were 91.6, 90.8 and 100% for the extracts BxbO, BxbR1 and BxbR2, respectively (Figure 4). The concentrations of 200 to 400 µg/mL in all extracts for both times of exposure significantly decreased the cell viability (*p* < 0.01). At the concentration of 100 µg/mL of BxbR2 extract, it was possible to observe proliferation in both the intervals: 24 and 48 h. In contrast, in cell cultures treated with 300 and 400 µg/mL of this extract, the percentages of viability were significantly lower for both 24 and 48 h (Figure 4).

This data allowed selecting the optimal non-cytotoxic concentrations of the extracts as 100 µg/mL, which was used in further experiments.

### 3.6. Hydrogen Peroxide-Induced Oxidative Stress and Survival

**Figure 5:**
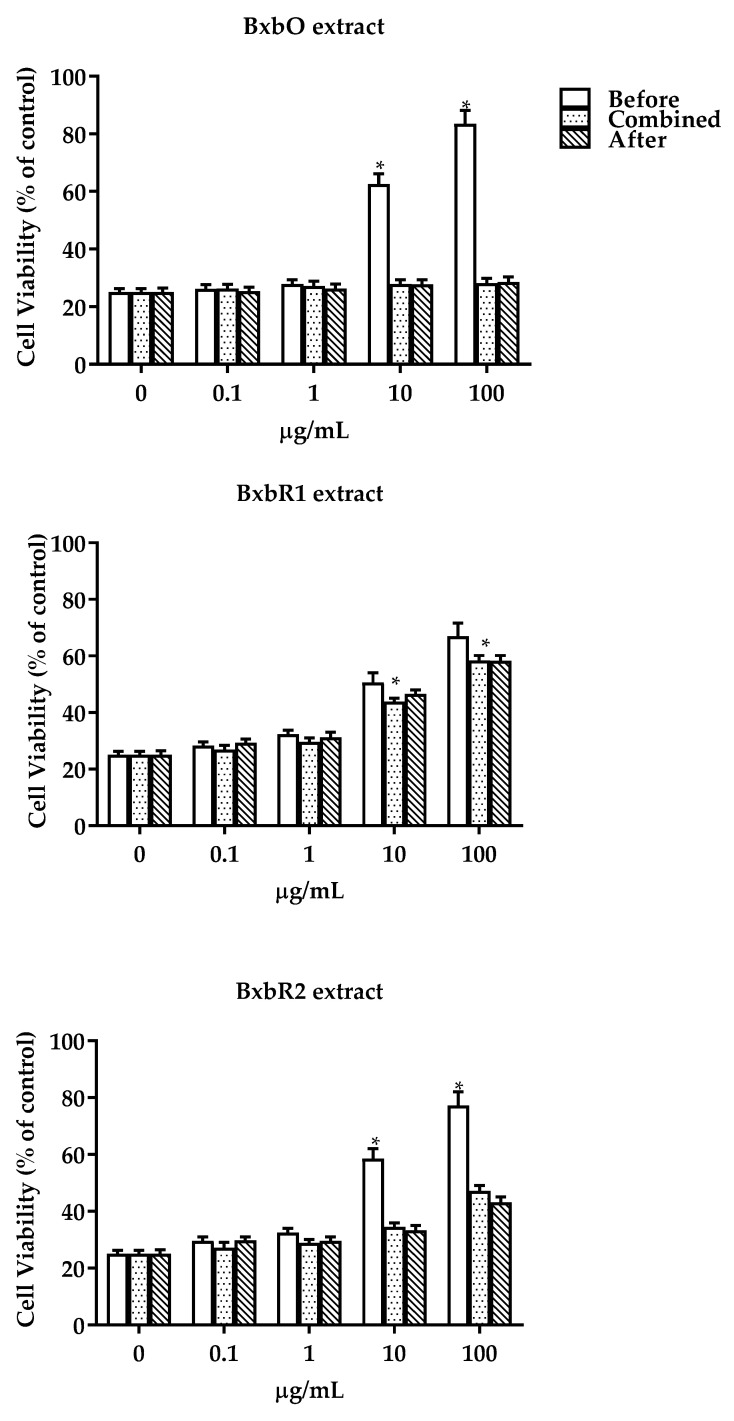
Effect of extracts from *B.* × *buttiana* on the cell viability. The percentage of survival of L929 cells treated with extracts in different conditions, before, combined and/or after exposition to H_2_O_2_ for 24 h was calculated as described in the Materials and Methods. The data are expressed as the mean ± SD (*n* = 20). * *p* < 0.001.

H_2_O_2_ as a precursor of various ROS was chosen as the oxidant reagent in this study. Various concentrations of H_2_O_2_ were used to determine the appropriate dose. L929 cells were treated with 1 mM hydrogen peroxide as a model of oxidative stress, and this caused a decrease in the cell viability by around 75% after 24 h of exposure. In Figure 5, the potential antioxidant effect of the extracts was tested before and after exposure to H_2_O_2_ for 3 h and combined with H_2_O_2_ for 24 h. In comparison, the extracts were effective in the cellular protection against oxidative stress before exposure to H_2_O_2_, resulting in a protection of 83.46, 66.96 and 77.21% for the extracts BxbO, BxbR1 and BxbR2, respectively. However, when the cells were exposed to extracts after or combined with H_2_O_2_ for 3 h, they did not protect the cells against the oxidative stress caused by H_2_O_2_ (Figure 5). In the BxbO and BxbR2 extracts, the best cell protection, expressed as cell viability values, was observed for treatment with 10 and 100 µg/mL before H_2_O_2_. In contrast, for the BxbR1 extract with the best cell protection, the viability values for the treatments of 10 and 100 µg/mL were combined and after H_2_O_2_ (Figure 5).

**Figure 6:**
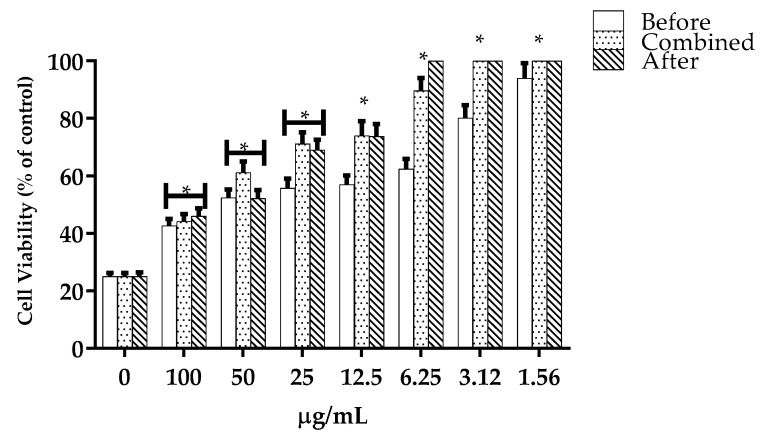
Effects of 2-Hydroxycinnamic acid on the cell viability. The percentage of survival of L929 cells treated with different amounts of 2-Hydroxycinnamic acid in different conditions, before, combined and/or after exposure to H_2_O_2_ for 24 h was calculated as described in the Materials and Methods. The data are expressed as the mean ± SD (*n* = 20). * *p* < 0.001.

Once the mode and amount of extract with the highest percentage of cell viability was established, we also compared the level of protection with the compounds present in the extracts. The results presented in Table 1 show that of all the different compounds found in the extracts BxbO, BxbR1 and BxbR2, where one of them was the phenolic compound, we found 2-Hydroxycinnamic acid. Another new test was carried out to verify which of these compounds could be responsible for the protection of the oxidative stress of the extracts (Figure 6). The treatment of L929 cells with 2-Hydroxycinnamic acid was shown to be effective in cell protection under the three different conditions. In the cultures of cells treated with 100 µg/mL of this compound before H_2_O_2_, the viability was significantly higher in relation to the control, with a percentage of 42.73% (Figure 6). In contrast, with the decrease in the concentration from 50 to 1.56 µg/mL, the viability was significantly increased (Figure 6). In cultures treated with 100 µg/mL of 2-Hydroxycinnamic acid combined or after H_2_O_2_, the viability percentages were 44.24 and 46.05%, respectively (Figure 6). Decreasing the compound dose from 50 to 6.25 µg/mL increased the cell viability (Figure 6). Under the conditions performed in this study, treatment with concentrations of 0.78 to 3.125 µg/mL, the viability percentages were greater than 100% (Figure 6).

**Figure 7:**
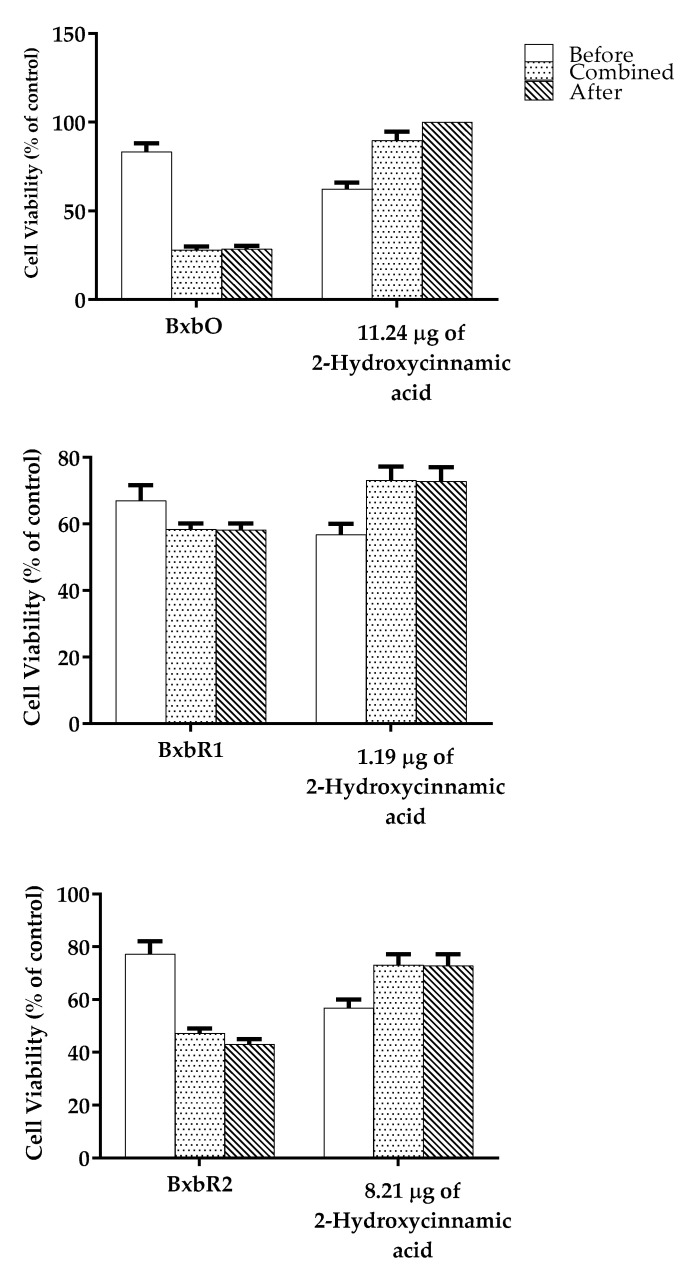
Comparison between *B*. × *buttiana* extracts and 2-Hydroxycinnamic acid on cell viability. The percentage of survival of L929 cells treated with 100 µg/mL of each extract and compared with culture cells with 11.24 µg/mL, 1.19 and 8.21 µg/mL of 2-Hydroxycinnamic acid for 24 h before exposition to H_2_O_2_ for 24 h was calculated as described in Material and Methods. Data are expressed as a mean ± SD (*n* = 20).

The groups of cells treated with 1 mg of *Bougainvillea* extracts contained 2-Hydroxycinnamic acid in the concentrations of 11.24, 1.90 and 8.21 µg for BxbO, BxbR1 and BxbR2, respectively. The different concentrations present in each extract were used to compare their effects with the total extract in cell cultures treated before, combined, or after H_2_O_2_ (Figure 7). The results obtained in cell cultures treated before H_2_O_2_ showed that 2-Hydroxycinnamic acid was not significant for the cytoprotective effect achieved for whole extracts. These results suggest that this treatment effect may be due to the action of other compounds.

### 3.7. In Silico Analysis of the Compounds

The results of the analysis on the biological effects of the compounds present in the extracts were obtained from PASSonline (Table 4). It was observed that the phenolic compounds and fatty acids presented a greater probability of acting as cytoprotective; however, the compound 2-Hydroxycinnamic acid was present in all three extracts and presented a higher probability of antioxidant and cytoprotective activity.

Derived from the previous analysis, an in silico analysis of the toxicological activity was performed in the OSIRIS Property Explorer program of the different compounds present in the three extracts. The results are coded in colors, as shown in Table 5. The properties without risk are represented in green color, those of moderate risk are represented in yellow color and those with high risk are represented in red color for undesirable effects such as mutagenicity, teratogenicity, irritability, and effects on the reproduction. The 2-Hydroxycinnamic acid compound shows zero risk of undesirable effects, and being present in the three extracts was decisive for its antioxidant and cytoprotective evaluation.

## 4. Discussion

The antioxidant potentiality of plant extracts was also extensively proven by using various assays in vitro [35]. The radical-scavenging assay and chelate transition metals have gained acceptance among researchers for their capacity to rapidly screen materials of interest. The phenolics derived from plants are well known as beneficial for human health due to their antioxidant activity, and they can act in different ways to protect cells from oxidative damage. Research found that micromolar concentrations of vitamin E and many polyphenols are effective against oxidative stress through the direct scavenging of reactive oxygen species, as shown in the cell culture model for the study of different diseases. The mechanism of action includes the prevention of reactive species formation, scavenging radicals or repairing damage to target molecules [36]. The estimation of total phenolic content is an important parameter to determine the amounts of antioxidants [21,22,32,33,34]. The amount of total phenolic contents in the extracts varied among extracts samples. These differences could be due to the different physicochemical properties of the phenolic components. In extracts of *Bougainvillea*, various phenolic compounds were obtained, and we investigated the antioxidant activity of extracts [21,22,33,34]. We have documented that *Bougainvillea* extracts have significant antioxidant capacity due to their phenolic compounds [22,32,33,34]. In the present study, the ability to inhibit DPPH production appeared to be significantly potent in all *Bougainvillea* extracts. With respect to the ORAC assay based on the delay of oxidation, the ORAC values of different extracts obtained in this study ranged from 7114.43 to 8105.38 µmol Trolox/g of dry extract, suggesting the extraction system used in this study was efficient at extracting potent antioxidant compounds.

The antioxidant activity of Bxb extract is thought to be related to its content of phenolic compounds. However, these molecules are unstable and could easily suffer degradation. Therefore, some parameters such as temperature, exposure to O_2_, and the time of extraction should be monitored during the extraction process to avoid the loss of biological activity as a consequence of the phenolic compound degradation [36]. In this study, the scavenging properties of Bxb extracts were tested in vitro using the NO scavenging method. The NO is mediated by an antioxidant compound through nitrogen atom donation, forming a stable compound. Although the Bxb extracts revealed an interesting antioxidant activity at higher concentrations, they were harmful to L929 cells in culture. In L929 cells treated with 1 mg/mL extract concentrations, the viability percentages were around 80%. Concentrations of 2 to 4 mg/mL of Bxb extracts were cytotoxic to L929 cells by around 30% after 24 and 48 h of exposure. This effect could be explained by a possible pro-oxidant property exerted by high concentrations of antioxidants. The phenolics compounds were capable of converting iron and cooper ions into their reduction forms which react with hydrogen peroxide to produce the highly toxic hydroxyl radicals [37,38]]. Factors that contribute to turning antioxidants into pro-oxidants included the concentrations of the antioxidants, nature of the neighboring molecules and availability of transitions metals [39,40]. Nitrile oxide is a compound synthesized by living organisms from the moment that nitric oxide synthase (NOS) becomes catalytically active, converting the amino acid L-arginine to NO and L-citrulline. When stimulated, human phagocytes can also produce this radical in greater quantities. It is a free radical that acts on a variety of biological processes, such as muscle relaxation, transmission, and immune regulation. While diffusing rapidly between and within cells, it is not reactive enough to attack DNA directly; however, it can react with the superoxide anion radical O_2_, and when exposed to air, it reacts with oxygen, forming NO_2_ [41,42].

Hydrogen peroxide alone is practically innocuous, and although it is not considered a free and important radical, it can easily diffuse through cell membranes and participate in reactions that produce the OH- radical in the presence of metals such as iron. H_2_O_2_ in vivo is generated by the dismutation of O_2_^−^ or by the β-oxidation of fatty acids [43]. All Bxb extracts at concentrations between 0.001 and 1 mg/mL were investigated regarding their protective effect using an in vitro oxidative stress model. For this, L929 cells were exposed to H_2_O_2_ for 3 h, and the extracts were added to the culture medium before or after H_2_O_2_ exposure. In addition, cells were also treated with a combination of Bxb extracts and H_2_O_2_ for 24 h. Hydrogen peroxide-induced oxidative stress concomitantly or after the extracts was lethal for L929 cells. H_2_O_2_ is a small uncharged molecule that could diffuse through cellular membranes easily [44]. This rapid transport to inside the cell could be responsible for the deleterious effect of H_2_O_2_ that acted faster than the cytoprotective phytochemicals added to the culture medium. The mitochondria that are sources of O_2_ are also rich in dismutase superoxide (SOD) that converts this anion radical into H_2_O_2_, and, thus, the hydrogen peroxide generated and partially eliminated by catalase, peroxidase glutathione and peroxidase linked to thioredoxin. However, this elimination has low efficiency, and a large part of the H_2_O_2_ is released from other regions of the cell.

Extracts added before H_2_O_2_ exposure were effective in protecting L929 cells from death, mainly with the BxbR2 extract. Preincubation with the extracts led to metabolic changes that resulted in cytoprotective effects against severe stress caused by H_2_O_2_. Phytochemicals can promote mild stress induction that elicits adaptative beneficial responses, thereby increasing protection against a further oxidative challenge. This process is known as hormesis and might contribute to explaining the results herein described [45,46].

Protection against oxidative stress occurs through two mechanisms [47]. First, the antioxidant functions through a direct antioxidant system, where the antioxidants are redox active with a lifespan and are sacrificed when they act on ROS. Due to this direct effect, antioxidants need to be regenerated so that they can curtail ROS levels. The second mechanism is an indirect antioxidant effect, which can trigger the self-defense mechanisms of the host cells to fight oxidative stress. Phenolic acids are divided into polyphenols, oligophenols and monophenols or simple phenolic compounds such as benzoic and cinnamic acids and their hydroxylated derivatives. Hydroxycinnamic acid derivatives are naturally occurring phenolic compounds that have an aromatic ring with a carbon chain, consisting of three carbons attached to the ring. These acids exist in plants, usually in the form of esters, such as chlorogenic acid or the ester of quinic acid. They are also found in the form of glycosides or linked to proteins and other cell wall polymers and, rarely, as free acids. Isomers of chlorogenic acid and caffeic acid are described with antioxidants [48,49]. These groups of phenolic acids have shown antioxidant properties. Although other characteristics also contribute to the antioxidant activity of phenolic acids and their esters, this is generally determined by the number of hydroxyls present in the molecule [50]. The presence of a second hydroxyl in the ortho or para position also increases the antioxidant activity. The sequestering effect of the hydroxyl radical appears to be directly related to the hydroxyl groups located in the para position in the aromatic ring.

In an in vivo model of the efficacy of 2-Hydroxycinnamic acid at a dose of 50 mg/kg body weight on oxidative stress in Winstar rats, a modest improvement in reactive oxygen species levels was demonstrated by induction with trichlorfon [51]. Therefore, it will be advisable to carry out studies in the future with other types of free radicals in an in vitro model in order to reduce the use of laboratory animals.

The in silico analysis was carried out through PASSonline, a platform that makes predictions about biological activities through the structure–activity relationship with an accuracy greater than 95% [30]. The chemical compounds 2-Hydroxycinnamic acid and 3-O-methyl-D-glucose are present in all three extracts; however, only 2-Hydroxycinnamic acid had high antioxidant and cytoprotective potential. The results obtained by the OSIRIS program on toxicity detected a greater number of molecules with a high risk of unwanted effects in the BxbO extract than in the other two extracts.

## 5. Conclusions

The production of phenolic compounds by plants is vast (about 8000 compounds have already been detected). These compounds are natural constituents that exert antioxidant action due to their chemical structure (a benzene ring with associated hydroxyls), and they can provide several biological benefits. Among all that has been discovered, research involving antioxidant agents in the *B*. × *buttiana* plant must continue, as they are of great importance for the pharmaceutical industry. With regard to the pharmaceutical field, the search continues for substances’ functional aspects in the fight against free radicals and all the possible dangers they pose to human health. According to our results, we comparatively investigated the total phenolic contents of BxbO, BxbR1 and BxbR2 extracts and tested their antioxidant and cytoprotective activities. These extracts exerted scavenging activity on NO and DPPH radicals, with a higher inhibitory extend detected in the DPPH assay. Other potential antioxidant activity, determined using ORAC assay, showed that *Bougainvillea* extracts are active radical scavengers. In the present study, the results indicated that BxbO, BxbR1 and BxbR2 extracts exert antioxidant and protective activities against oxidative stress induced by H_2_O_2_ on L929 cells. The results obtained showed that these extracts exert a protective action by decreasing cell death and by inhibiting around of 64% of NO radical activity, suggesting that these phenolic contents may be useful for those oxidative stress-related degenerative diseases. Follow-up studies that isolate and test individual constituents in the extracts and different combination studies may help to determine the specific health-protective effects of each constituent.

## Figures and Tables

**Table 1:** Comparison of the compounds found in *Bougainvillea* extract.

Class	Compounds	R.T. (min)	Area%		
			BxbO	BxbR1	BxbR2
Pyran	3,5-Dihydroxy-6-methyl-2,3-dihydro-4H-pyran-4-one	10.293			5.867
Phenol compounds	2-Hydroxycinnamic acid	11.306	11.240	1.190	8.206
Benzofuran	2,3-Dihydro-1-benzofuran	11.621			15.469
Phenol compounds	2-Methoxy-4-vinylphenol	11.925		0.224	5.588
Carbohydrate	3-O-methyl-D-glucose	16.292	11.192	92.135	47.924
Fatty acid	Tetradecanoic acid	17.369	10.765		
Hydrocarbon	1-Nonadecene	17.750	10.726		
Fatty ester	Methyl palmitate	19.096			5.535
Fatty acid	*n*-Hexadecanoic acid	19.445	11.522	0.760	
Fatty ester	Hexadecanoic acid, ethyl ester	19.656		1.171	5.654
Fatty ester	Isopropyl palmitate	20.082	10.985		
Fatty ester	9,12-Octadecadienoic acid, ethyl ester	21.246		1.933	5.757
Fatty ester	Ethyl (9Z,12Z,15Z)-octadeca-9,12,15-trienoate	21.311		2.587	
Ester	Diisopropyl maleate	21.967	11.258		
Phthalate ester	1,2-Benzenedicarboxylic acid, diisooctyl ester	25.692	11.237		
Terpene	Squalene	29.981	11.075		

BxbO (Orange), BxbR1 (Rose1) and BxbR2 (Rose2).

**Table 2:** Different total phenolic contents in *Bougainvillea* extracts.

Extract	Total Phenolic Contents, mgGA */dry Extract
BxbO	27.43 ± 0.03
BxbR1	29.55 ± 0.05
BxbR2	32.48 ± 0.08

Data are expressed as the mean of triplicate ± SD. * Gallic Acid (GA). BxbO (Orange), BxbR1 (Rose1) and BxbR2 (Rose2).

**Table 3:** Antioxidant activity of *Bougainvillea* extracts.

Extract	DPPH µMTE/100 g Dry Extract	ORAC Value µM Trolox Equivalent TE/g
BxbO	1478.20 ± 100.80	7114.43 ± 781.48
BxbR1	1684.63 ± 146.37	8100.45 ± 798.85
BxbR2	1802.54 ± 158.46	8105.38 ± 787.92

Values represent means (*n* = 3) ± SD. BxbO (Orange), BxbR1 (Rose1) and BxbR2 (Rose2).

**Table 4:** PASSonline activities.

Extracts	Compounds	Antioxidant		Cytoprotective	
		Pa	Pi	Pa	Pi
BxbR2	3,5-Dihydroxy-6-methyl-2,3-dihydro-4H-pyran-4-one	0.587	0.005	0.652	0.013
BxbO, BxbR1, BxbR2	2-Hydroxycinnamic acid	0.523	0.006	0.679	0.008
BxbR2	2,3-Dihydro-1-benzofuran	0.176	0.073	0.482	0.068
BxbR1, BxbR2	2-Methoxy-4-vinylphenol	0.459	0.008	0.735	0.004
BxbO, BxbR1, BxbR2	3-O-methyl-D-glucose	-	-	0.352	0.130
BxbO	Tetradecanoic acid	0.222	0.045	0.712	0.004
BxbO	1-Nonadecene	0.282	0.027	0.655	0.013
BxbR2	Methyl palmitate	0.210	0.050	0.701	0.005
BxBO, BxbR1	*n*-Hexadecanoic acid	0.222	0.045	0.712	0.004
BxbR1, BxbR2	Hexadecanoic acid, ethyl ester	0.201	0.055	0.715	0.004
BxbO	Isopropyl palmitate	0.227	0.043	0.654	0.013
BxbR1, BxbR2	9,12-Octadecadienoic acid, ethyl ester	0.285	0.026	0.720	0.004
BxbR1	Ethyl (9Z,12Z,15Z)-octadeca-9,12,15-trienoate	0.320	0.020	0.704	0.005
BxbO	Diisopropyl maleate	0.378	0.014	0.618	0.024
BxbO	1,2-Benzenedicarboxylic acid, diisooctyl ester	0.174	0.075	0.512	0.059
BxbO	Squalene	0.657	0.004	0.552	0.047

Pa—probability of being active, Pi—probability of being inactive., BxbO (Orange), BxbR1 (Rose1) and BxbR2 (Rose2).

**Table 5:** Toxicity risk by OSIRIS Property Explorer.

Extracts	Compounds	M	T	I	R.E.
BxbR2	3,5-Dihydroxy-6-methyl-2,3-dihydro-4H-pyran-4-one	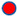	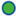	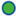	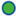
BxbO, BxbR1, BxbR2	2-Hydroxycinnamic acid	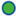	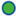	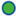	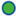
BxbR2	2,3-Dihydro-1-benzofuran	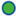	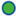	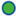	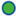
BxbR1, BxbR2	2-Methoxy-4-vinylphenol	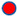	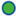	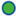	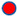
BxbO, BxbR1, BxbR2	3-O-methyl-D-glucose	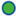	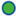	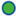	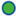
BxbO	Tetradecanoic acid	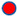	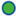	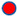	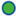
BxbO	1-Nonadecene	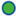	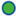	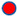	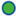
BxbR2	Methyl palmitate	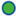	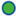	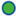	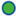
BxbO, BxbR1	*n*-Hexadecanoic acid	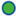	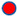	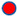	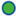
BxbR1, BxbR2	Hexadecanoic acid, ethyl ester	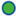	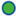	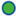	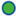
BxbO	Isopropyl palmitate	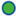	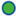	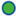	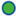
BxbR1, BxbR2	9,12-Octadecadienoic acid, ethyl ester	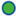	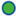	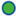	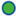
BxbR1	Ethyl (9Z,12Z,15Z)-octadeca-9,12,15-trienoate	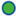	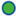	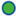	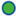
BxbO	Diisopropyl maleate	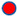	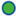	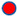	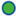
BxbO	1,2-Benzenedicarboxylic acid, diisooctyl ester	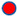	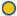	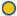	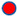
BxbO	Squalene	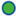	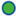	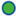	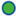

M: Mutagenic, T: Tumorigenic, I: Irritability, R.E. Reproductive effect. 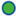: Risk free, 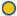: Medium risk, 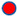: High risk., BxbO (Orange), BxbR1 (Rose1) and BxbR2 (Rose2)

## Data Availability

The data are contained within the article.
